# Elevated blood lead and cadmium levels associated with chronic infections among non-smokers in a cross-sectional analysis of NHANES data

**DOI:** 10.1186/s12940-016-0113-4

**Published:** 2016-02-11

**Authors:** Whitney S. Krueger, Timothy J. Wade

**Affiliations:** RTI Health Solutions, 3040 Cornwallis Road, Post Office Box 12194, Research Triangle Park, NC 27709-2194 USA; Oak Ridge Institute for Science and Education, Oak Ridge, TN 37831 USA; United States Environmental Protection Agency, Office of Research and Development, National Health & Environmental Effects Research Laboratory, Environmental Public Health Division, Chapel Hill, NC 27514 USA

**Keywords:** Helicobacter pylori, Toxoplasma, Hepatitis B, Nutrition surveys, Seroprevalence, Heavy metals, Immune system, Immunomodulation

## Abstract

**Background:**

Experimental animal studies, *in vitro* experiments, and clinical assessments have shown that metal toxicity can impair immune responses. We analyzed data from a United States representative National Health and Nutrition Examination Survey (NHANES) to explore associations between chronic infections and elevated blood concentrations of lead and cadmium among non-smoking NHANES participants.

**Methods:**

NHANES data from 1999 to 2012 were examined and weighted to represent the United States population. Multivariable logistic regression was used to estimate adjusted odds ratios (AOR) and 95 % confidence intervals (CI) for heavy metal associations with seropositivity for *Helicobacter pylori*, *Toxoplasma gondii*, and Hepatitis B virus (HBV) infections.

**Results:**

Available 2-year survey cycles for infection seroprevalence varied by pathogen, from 1 to 7 cycles. Available sample size, disease seroprevalence, and participant age range also varied by pathogen of interest. After controlling for demographic characteristics and general health condition, an elevated blood lead level above the survey population median was significantly associated with seropositivity for all three pathogens (AORs = 1.2–1.5). In addition, an elevated blood cadmium level above the median was significantly associated with HBV (AOR = 1.5; 95 % CI = 1.2–2.0) and *H. pylori* (AOR = 1.5; 95 % CI = 1.2–1.7) seropositivity. Age-specific analyses for *H. pylori* and *T. gondii* indicated stronger associations among children under 13 years of age, particularly for lead exposure and *H. pylori* seropositivity, and weaker associations among those over 35 years of age.

**Conclusions:**

The results of this cross-sectional human health survey suggest that the immunological effects of lead and cadmium toxicity may be associated with an increased susceptibility to chronic infections.

**Electronic supplementary material:**

The online version of this article (doi:10.1186/s12940-016-0113-4) contains supplementary material, which is available to authorized users.

## Background

Human exposure to heavy metals can impair innate and humoral immune responses and lead to an increased susceptibility to infections as well as the development of autoimmune diseases [1, 2]. Immunomodulatory effects include modifying inflammatory reactions, increasing cytotoxic responses, and altering the number of circulating lymphocytes, natural killer (NK) cells, and memory cells [3–7]. Prenatal exposures to arsenic and cadmium have been shown to impact genes that may underlie altered infectious disease susceptibility [8]. While direct immunotoxicity of heavy metals has been examined through animal studies, *in vitro* experiments, clinical assessments, and occupationally-exposed cohort studies [7, 9, 10], few large-scale human epidemiological studies have been performed to assess the adverse immunological effects of heavy metal exposure. In this analysis, we examined associations between three common chronic infections: *Helicobacter pylori, Toxoplasma gondii,* and Hepatitis B virus (HBV) and two ubiquitous heavy metals: Lead (Pb) and Cadmium (Cd).

Naturally found in the earth’s crust, lead is used to make batteries, ammunition, pipes, and roofing materials [11]. In the past, it was also added to paint and gasoline. Humans are exposed to lead by inhaling contaminated air, dust, or lead dust, as well as ingesting contaminated soil, water, food, paint chips, and painted toys. Construction sites [12] and hazardous waste sites [13] present occupational exposure risks. Absorption of lead in the body depends on several factors, including nutrition status, health, and age; adults typically absorb up to 10 % of ingested lead while children can retain up to 50 % [11]. Absorbed lead accumulates in mineralizing and soft tissues. Unborn children are at the greatest risk of adverse health effects associated with lead exposure, including low birth weight, premature birth, and abortion. In young children, lead exposure has been associated with decreased intelligence, slowed growth, and hearing problems. Lead has not been shown to be a human carcinogen, but exposure to high levels of lead can result in kidney and brain damage [11].

Also naturally found in the earth’s crust, cadmium is a byproduct of smelting and is used in metal plating as well as in making batteries, pigments, and plastics. Cadmium is released into soil, water, and air via mining/refining practices, fertilizer application, fossil fuel combustion, and waste disposal, where it can accumulate in aquatic organisms and agricultural crops [14]. Humans are primarily exposed to cadmium through smoking tobacco and eating contaminated vegetables and grains. Occupational exposure risks through inhalation or accidental ingestion are also of concern. Up to 10 % of ingested cadmium enters the body through the digestive tract [15]. The body can convert cadmium to a non-harmful form; however, at high levels the liver and kidneys can become overloaded. As one of the most toxic heavy metals [16], acute toxicity induces vomiting and diarrhea. Chronic low-level exposures can result in kidney damage and brittle bones [17]. The U.S. Environmental Protection Agency (EPA) has designated cadmium in the B1 classification as a probable human carcinogen, citing studies linking lung cancer and workers who inhaled cadmium [18].

We analyzed data from the nationally representative National Health and Nutrition Examination Survey (NHANES) to explore associations between *H. pylori*, *T. gondii* and HBV seropositivity and elevated blood concentrations of lead and cadmium among non-smoking NHANES participants.

## Methods

Seven 2-year cycles of continuous NHANES data (1999–2012) [19] were examined. Data were collected using a complex sampling survey design; therefore, approaches recommended by the U.S. Centers for Disease Control and Prevention (CDC) for SAS survey analysis procedures were followed and appropriate sample weights according to the NHANES Analytic and Reporting Guidelines were applied [20].

### Seroprevalence data

Available serology results for *H. pylori* (1999–2000)*, T. gondii* (1999–2004, 2009–10)*,* and HBV (1999–2012) were examined. For *H. pylori*, sera from eligible participants ≥3 years old were tested by the CDC for immunoglobulin G (IgG) antibodies using an enzyme-linked immunosorbent assay (ELISA) from Wampole Laboratories (Cat #446404, Carter Wallace, Inc., Cranbury, NJ). A dichotomous seropositivity cut-off was provided by the CDC, in which an optical density at 450 nm (OD_450_) ≥1.1 = positive and OD_450_ < 1.1 = negative [21]. For *T. gondii*, available serum from eligible participants 6–49 years old (1999–2004) and ≥6 years old (2009–10) were tested by the CDC for IgG antibodies using enzyme immunoassays (EIAs) (Bio-rad, Hercules, CA). A dichotomous seropositivity cut-off was provided by the CDC (1999–2004: IgG ≥10 IU/mL = positive; IgG <10 IU/mL = negative; 2009–2010: IgG ≥33 IU/mL = positive; IgG <33 IU/mL = negative).

Only naturally-acquired HBV infections were considered in this analysis. The commercially-available Ortho HBc ELISA Test System (Ortho-Clinical Diagnostics, Raritan, NJ), a qualitative ELISA, was used by the CDC to detect total antibodies against hepatitis B core (HBc) antigen. Anti-HBc is present in the serum of HBV-infected individuals and is considered an accurate serological marker of acute, chronic, or resolved HBV infections, but not a marker of vaccine-induced immunity. Participants who had received at least 1 dose of the 3-dose HBV vaccine series, as well as those with unknown vaccine status, were excluded from the analysis. In addition, participants seronegative for anti-HBc but seropositive for antibodies against the HBV surface antigen, a marker for both natural and vaccine-induced immunity, were also excluded from the analysis. NHANES participants ≥6 years old were tested for HBV seroprevalence from 1999 to 2012. NHANES reported HBV serology results dichotomously as positive or negative.

### Blood lead and cadmium levels

For the 1999–2002 survey years, lead and cadmium were simultaneously measured in whole blood by atomic absorption spectrophotometry [22–24] with quantifications based on the measurement of light absorbed at 228.8 nm and 283.3 nm, respectively, by ground-state atoms of cadmium and lead from either an electrodeless discharge lamp (EDL) or by a hollow cathode lamp (HCL) source [25]. For the remaining survey years (2003–2012), whole blood lead and cadmium concentrations were determined by inductively coupled plasma mass spectrometry (ICP-MS), a multi-element analytical technique based on quadrupole ICP-MS technology [26]. The lower detection limits for lead were 0.30 μg/dL for NHANES 1999–2004 and 0.25 μg/dL for 2005–2012. For cadmium, the limit of detection (LOD) was 0.30 μg/dL for NHANES 1999–2002, 0.20 μg/dL for 2003–2010, and 0.16 μg/dL for 2011–2012. Results below the LOD were replaced with a value equal to $LOD/\sqrt{2}$.

Whole blood concentrations of lead and cadmium were assessed by several exposure measurements, including weighted geometric means (GM), levels greater than the study population median, each 2-fold increase (doubling) in concentration, as well as by categories to assess potential nonlinear associations. Quartiles were used for lead, but for cadmium the distribution was skewed due to samples below the limit of detection, so tertiles were used to prevent overlapping values. Metal exposures were stratified by seroreactivity and associations between elevated blood concentrations and seropositivity were examined. Weighted linear correlations between log-transformed lead and cadmium concentrations were measured using Pearson’s *r*.

### Data analysis

All analyses were conducted using SAS v9.3 (SAS, Inc., Cary, NC, USA). Because cigarette smoking is highly correlated with toxic heavy metal exposures [27], we restricted our analyses to a domain of non-smokers, defined as having a serum cotinine level <10 ng/mL) [28]. Differences in seroprevalence by participants’ demographics were evaluated using the Rao-Scott chi-square test for unadjusted bivariate comparisons. The authors selected covariates of interest *a priori,* based on previous reports and biologic plausibility, to examine associations with seropositivity, including NHANES cycle, age, gender, race/ethnicity, family income (</≥$20,000), country of birth origin (born in the United States or elsewhere), self-reported general health condition (excellent/very good, good, fair/poor), source of tap water in home (municipal or well), crowded housing (total number of individuals living in a household divided by number of rooms in the home, excluding bathroom and kitchen), and ever using a needle to take street/illicit drugs. Because of its role in the replication of bacteria and viruses and its potential impact in the uptake of divalent metals such as lead and cadmium, we also considered serum iron as a potentially important confounding variable [29].

Associations between elevated lead and cadmium blood levels and seropositivity for each pathogen were examined with unadjusted and adjusted logistic regression models to ascertain Rao-Scott chi-square odds ratio (OR) estimates and 95 % confidence intervals (CI).

Multivariable logistic regression was used to account and control for key demographic characteristics and known risk factors, with one model fitted for each serology outcome that included lead or cadmium individually. Covariates with p values <0.2 in a simple logistic model were considered for inclusion in multivariable models. Multivariable models were selected using stepwise backwards elimination of covariates with p values >0.05, with the highest corresponding p-value removed at each step while minimizing the Akaike information criterion (AIC). Collinearity of lead and cadmium levels was confirmed to be insignificant by evaluating the Pearson correlation coefficient, variation inflation factor, and condition number for each model.

Trend tests were performed by regressing the outcome of interest on categorical median concentrations in each percentile (quartiles for lead and tertiles for cadmium) and including all of the same model covariates. Departure from additivity for the joint association of elevated blood lead and cadmium concentrations was assessed by estimating the relative excess risk due to interaction (RERI) and 95 % confidence intervals using the delta method described by Hosmer and Lemeshow [30]. The products of blood lead and cadmium concentrations were added to the logistic regression models to evaluate interaction between the two exposures of interest on a multiplicative scale.

To examine whether the associations differed by age, we stratified the analysis by three age groups (under 13 years of age, 13-35 years of age and over 35 years of age) for *H. pylori* and *T. gondii*. Age stratification was not considered for HBV because too few children (only 2 were under 13 years of age) were seropositive.

## Results

### Helicobacter pylori

**Fig. 1:**
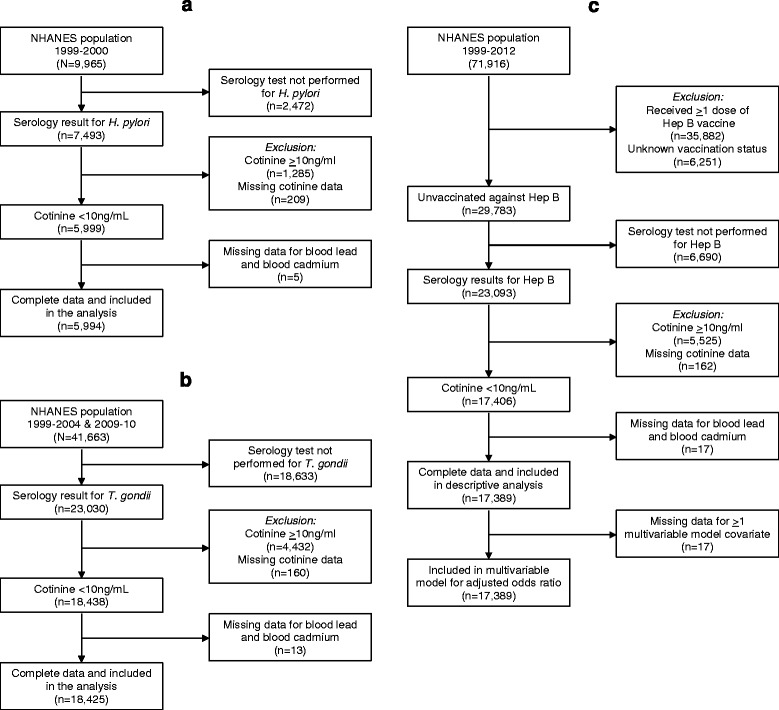
Flow charts illustrating derived study populations for each pathogen of interest: **a**) *Helicobacter Pylori*; **b**)*Toxoplasma gondii* **c**) Hepatitis B Virus. NHANES 1999-2012

**Fig. 2:**
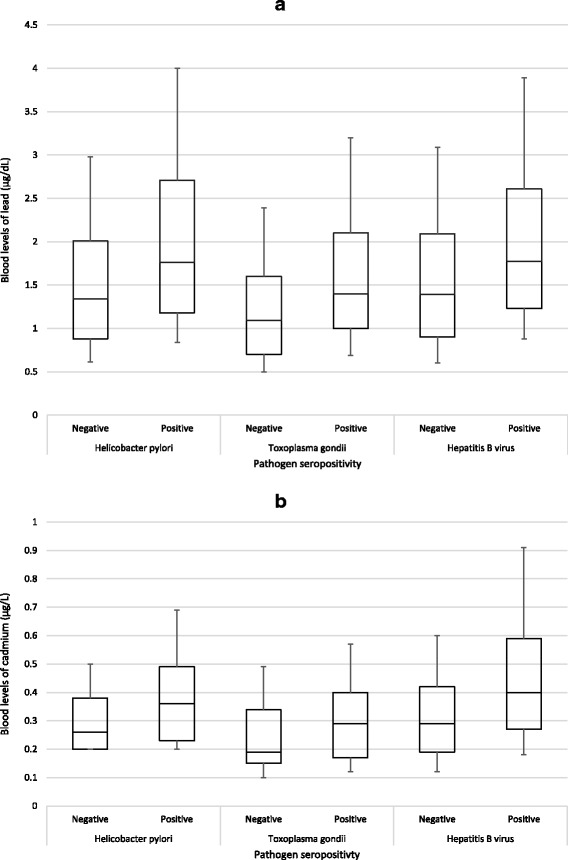
Box plots of 10^th^, 25^th^, 50^th^, 75^th^, and 90^th^ percentiles for blood levels of lead (panel **a**) and cadmium (panel **b**) by pathogen seropositivity among non-smoking NHANES participants, 1999–2012

**Fig. 3:**
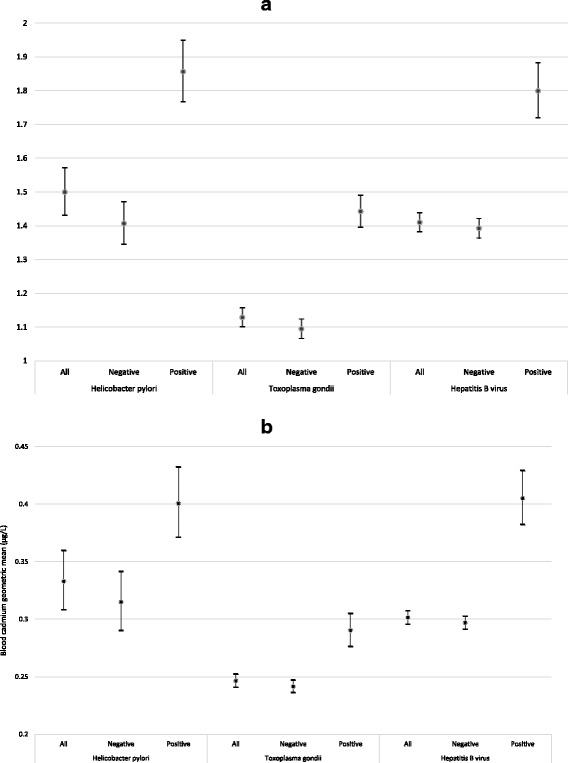
Geometric means and 95 % confidence intervals of blood metal of lead (panel **a**) and cadmium (panel **b**) by pathogen seropositivity among non-smoking NHANES participants, 1999–2012

Exposure and *H. pylori* serology data were available for 5994 non-smoking participants ≥3 years old from a single NHANES cycle (1999–2000) (Fig. 1a). Overall seroprevalence for IgG antibodies against *H. pylori* was 23.1 %. Seroreactivity significantly varied by age, race, family income level, country of birth origin, general health condition, and crowded housing (Table 1). The blood lead GM concentration for all participants was 1.50 (95 % CI = 1.43–1.57), with 0.8 % of results below the LOD; the GM for blood cadmium was 0.33 (95 % CI = 0.31–0.36), with 35.1 % below the LOD (Table 2). In examining the association between seropositivity and blood concentrations of lead and cadmium (Figs. 2 and 3), the adjusted odds ratios associated with a 2-fold increase in blood lead and cadmium were 1.22 (95 % CI = 1.12–1.34) and 1.37 (95 % CI = 1.17–1.62), respectively (Table 3). There were significant trends (*p* < 0.0001) in increasing seroprevalence by percentiles of lead and cadmium (Table 3). Lead and cadmium concentrations were only slightly correlated (Pearson’s *r* = 0.26), and no evidence was found to suggest additive or multiplicative interaction between lead and cadmium exposure in association with *H. pylori* seropositivity (Table 4).

**Table 1:** Weighted seroprevalence by participant characteristics among nonsmokers, NHANES 1999–2012

Characteristic	*H. pylori*			*T. gondii*			Hepatitis B virus		
	*N*	Positive (%)^a^	*P* ^b^	*N*	Positive (%)^a^	*P* ^b^	*N*	Positive (%)^a^	*P* ^b^
*Total*	5994	23.1		18,425	11.0		17,389	4.9	
NHANES cycle years									
1999–2000	5994	23.1	–	4084	13.5	0.0001	2926	5.0	0.8283
2001–2002	–	–		4669	8.0		2955	4.5	
2003–2004	–	–		4089	9.3		2420	5.7	
2005–2006	–	–		–	–		2157	5.3	
2007–2008	–	–		–	–		2507	4.3	
2009–2010	–	–		5583	12.8		2466	5.0	
2011–2012	–	–		–			1958	4.9	
Age (years)									
≤ 13	1721	7.2	<0.0001	5127	3.5	<0.0001	613	0.4	<0.0001
14–40	2307	20.2		9252	10.5		4788	2.9	
≥ 41	1966	33.4		4046	16.2		11,988	5.9	
Gender									
Male	2785	22.7	0.5279	8529	11.5	0.1633	8171	5.5	0.0185
Female	3209	23.5		9896	10.5		9218	4.5	
Race									
Non-Hispanic White	1912	14.6	<0.0001	6320	9.0	<0.0001	8365	2.6	<0.0001
Non-Hispanic Black	1300	37.2		4313	11.7		3124	13.4	
Mexican American	2586	45.2		6967	17.2		5083	4.3	
Other	196	31.2		825	12.4		817	27.4	
Family income									
≥ $20,000	2991	18.3	<0.0001	10,997	9.6	<0.0001	10,661	4.0	<0.0001
< $20,000	2650	31.5		6725	14.3		6027	7.4	
Country of birth origin									
United States	4675	17.6	<0.0001^c^	14,417	7.7	<0.0001	12,919	3.2	<0.0001
Other	1314	53.4		4002	27.3		4459	14.0	
General health condition									
Excellent/very good	3190	17.2	<0.0001	10,634	8.2	<0.0001	7622	3.8	<0.0001
Good	1855	28.0		5452	13.7		5829	5.6	
Fair/poor	943	42.0		2330	20.9		3925	7.5	
Source of tap water									
Municipal	5306	22.9	0.9746	16,294	10.9	0.9501	13,218	5.4	<0.0001
Public/private well	589	23.0		1768	11.0		1962	2.6	
Crowded housing^d^									
≤ 1 person/room	4109	20.9	<0.0001	12,739	10.1	<0.0001	12,872	4.8	0.0091
> 1 person/room	1806	34.2		5490	15.7		2401	6.5	
Injected drug user									
No/unknown	–	–	–	18,377	8.4	0.5850	17,294	4.8	<0.0001
Yes	–	–		48	11.0		95	18.5	

^a^Weighted percentages

^b^Rao-Scott chi-square test used

^c^Unweighted Wald chi-square used

^d^Calculated as total number of individuals living in a household divided by number of rooms in the home (excluding bathroom and kitchen)

**Table 2:** Distribution of blood levels for lead and cadmium (in μg/mL) among non-smoking NHANES participants, 1999–2012

Survey years	Pathogen	*N*	Blood lead					Blood cadmium				
			Weighted Geometric Mean (95 % CI)	25^th^ P	50^th^ P	75^th^ P	% below LOD^a^	Weighted Geometric Mean (95 % CI)	25^th^ P	50^th^ P	75^th^ P	% below LOD^b^
1999–2000	*Helicobacter pylori*	5994	1.50 (1.43–1.57)	0.94	1.43	2.18	0.80	0.33 (0.31–0.36)	0.20	0.29	0.40	35.1
	Negative	4073	1.41 (1.34–1.47)	0.88	1.34	2.01	0.96	0.31 (0.29–0.34)	0.20	0.26	0.38	39.6
	Positive	1921	1.86 (1.77–1.95)	1.18	1.76	2.71	0.26	0.40 (0.37–0.43)	0.23	0.36	0.49	20.4
1999–2004, 2009–2010	*Toxoplasma gondii*	18,425	1.13 (1.10–1.16)	0.72	1.10	1.69	0.95	0.25 (0.24–0.25)	0.15	0.20	0.38	44.7
	Negative	16,449	1.09 (1.07–1.12)	0.70	1.09	1.60	1.01	0.24 (0.24–0.25)	0.15	0.19	0.34	46.4
	Positive	1976	1.44 (1.40–1.49)	1.00	1.40	2.10	0.47	0.29 (0.28–0.31)	0.17	0.29	0.40	30.4
1999–2012	Hepatitis B virus	17,389	1.41 (1.38–1.44)	0.90	1.40	2.10	0.51	0.30 (0.30–0.31)	0.20	0.30	0.44	26.4
	Negative	16,231	1.39 (1.36–1.42)	0.90	1.39	2.09	0.53	0.30 (0.29–0.30)	0.19	0.29	0.42	14.7
	Positive	1158	1.80 (1.72–1.88)	1.23	1.77	2.61	0.18	0.41 (0.38–0.43)	0.27	0.40	0.59	27.1

*LOD* limit of detection

^a^LOD = 0.30 (1999–2004), 0.25 (2005–2012)

^b^LOD = 0.30 (1999–2002). 0.20 (2003–2010), 0.16 (2011–2012)

**Table 3:** Seropositivity associated with blood lead and cadmium levels, for each two-fold increase and across percentiles, among non-smoking NHANES participants, 1999–2012. All subjects

Heavy metal	*H. pylori*		*T. gondii*		Hepatitis B virus	
	Positive (Weighted %)	AOR (95 % CI)^a,b^	Positive (Weighted %)	AOR (95 % CI)^a,c^	Positive (Weighted %)	AOR (95 % CI)^a,d^
Per doubling of blood lead	23.1	**1.22 (1.12–1.34)** ^**e**^	11.0	**1.19 (1.12–1.28)**	4.9	**1.19 (1.08–1.32)**
Blood lead concentration (μg/dL)						
Quartile 1	12.7	Ref	5.6	Ref	2.2	Ref
Quartile 2	19.9	1.31 (0.98–1.75)	8.7	1.20 (0.93–1.54)	4.0	**1.41 (1.04–1.93)**
Quartile 3	24.3	**1.39 (1.13–1.72)**	12.8	**1.53 (1.22–1.91)**	6.1	**1.79 (1.31–2.43)**
Quartile 4	34.1	**1.69 (1.33–2.15)**	16.5	**1.56 (1.28–1.90)**	7.1	**1.70 (1.23–2.35)**
*p for trend*	<0.0001		<0.0001		0.0077	
Per doubling of blood cadmium	23.1	**1.37 (1.17–1.62)**	11.0	1.06 (0.96–1.18)	4.9	**1.38 (1.23–1.55)**
Blood cadmium concentration (μg/L)						
Tertile 1	13.4	Ref	6.9	Ref	2.8	Ref
Tertile 2	22.1	**1.51 (1.16–1.97)**	9.7	**1.34 (1.03–1.74)**	3.8	1.09 (0.84–1.43)
Tertile 3	30.5	**1.48 (1.25–1.76)**	14.4	**1.32 (1.03–1.69)**	7.7	**1.72 (1.32–2.25)**
*p for trend*	<0.0001		0.1438		<0.0001	

^a^Multivariable logistic regression used with survey procedures

^b^Adjusted for age, gender, race/ethnicity, country of birth origin, family income, self-reported general health condition, tap water source, and household crowding

^c^Adjusted for NHANES cycle, age, gender, race/ethnicity, country of birth origin, family income, self-reported general health condition, and household crowding

^d^Adjusted for age, gender, race/ethnicity, country of birth origin, family income, self-reported general health condition, and use of illicit/street injected drugs

^e^Bolded font denotes statistically significant (α < 0.05)

**Table 4:** Effect of interaction between blood lead and blood cadmium levels on odds of *H. pylori* seropositivity, NHANES 1999–2000

Blood lead levels	Blood cadmium levels				
	<Median		≥Median		AOR (95 % CI) within Pb strata
	N (Weighted %)	AOR (95 % CI)	N (Weighted %)	AOR (95 % CI)	
<Median	1662 (11.6)	1.0	1074 (23.7)	1.38 (1.03–1.97)	1.20 (0.88–1.64)
				*P* = 0.0337	*P* = 0.2597
≥Median	1436 (22.1)	1.43 (1.10–1.87)	4073 (35.2)	1.63 (1.27–2.10)	1.27 (1.02–1.57)
		*P* = 0.0082		*P* = 0.0002	*P* = 0.0313
AOR (95 % CI) within Cd strata		1.32 (0.97–1.79)		1.27 (0.98–1.65)	
		*P* = 0.0795		*P* = 0.0689	

Measure of interaction on additive scale: RERI (95 % CI) = -0.21 (−0.74–0.32)

Measure of interaction on multiplicative scale: ratio of ORs (95 % CI) = 0.82 (0.56–1.21); *P* = 0.3183

AORs are adjusted for age, gender, race/ethnicity, country of birth origin, family income, self-reported general health condition, tap water source, and household crowding

Positive associations between metal exposure and *H. pylori* seropositivity were stronger among children under 13 years old. Children in the highest quartile of lead exposure had markedly higher rates of *H. pylori* seropositivity (Table 5, OR = 21.2, 95 % CI = 8.32–53.8). The strength of the associations for both lead and cadmium declined among those aged 13–35 years. Among those over 35 years old, associations were not statistically significant (Additional file 1: Tables S1 and S2).

**Table 5:** Seropositivity associated with blood lead and cadmium levels, for each two-fold increase and across percentiles, among non-smoking NHANES participants, 1999–2012. Children under 13 years of age^a^

Heavy metal	*H. pylori*		*T. gondii*	
	Positive (Weighted %)	AOR (95 % CI)^b,c^	Positive (Weighted %)	AOR (95 % CI)^b,d^
Per doubling of blood lead	7.3	**2.61 (1.85–3.67)** ^**e**^	3.5	1.12 (0.84–1.49)
Blood lead concentration (μg/dL)				
Quartile 1	0.8	Ref	1.8	Ref
Quartile 2	4.5	**5.26 (1.92–14.4)**	3.0	1.46 (0.57–3.73)
Quartile 3	5.8	**4.75 (1.61–14.0)**	3.0	1.17 (0.46–2.95)
Quartile 4	15.5	**21.2 (8.32–53.8)**	5.7	1.82 (0.75–4.43)
*p for trend*	<0.0001		0.1825	
Per doubling of blood cadmium	7.3	**2.02 (1.29–3.15)**	3.5	1.10 (0.74–1.63)
Blood cadmium concentration (μg/L)				
Tertile 1	5.5	Ref	2.1	Ref
Tertile 2	9.9	**2.75 (1.30–5.83)**	4.1	1.46 (0.63–3.36)
Tertile 3	10.5	**2.39 (1.35–4.20)**	4.2	1.11 (0.48–2.56)
*p for trend*	0.0005		0.8214	

^a^Only two children under 13 were positive for Hepatitis B so it was not considered

^b^Multivariable logistic regression used with survey procedures

^c^Adjusted for age, gender, race/ethnicity, country of birth origin, family income, self-reported general health condition, tap water source, and household crowding

^d^Adjusted for NHANES cycle, age, gender, race/ethnicity, country of birth origin, family income, self-reported general health condition, and household crowding

^e^Bolded font denotes statistically significant (α < 0.05)

### Toxoplasma gondii

For *T. gondii*, 18,425 non-smoking participants ≥6 years old had complete data available from 4 NHANES cycles (1999–2004 and 2009–10) (Fig. 1b). The seroprevalence for IgG antibodies against *T. gondii* among the study population was 11.0 %. Seroreactivity against *T. gondii* significantly varied by NHANES cycle, age, race, family income level, country of birth origin, general health condition, and crowded housing (Table 1). The blood lead GM concentration for all participants was 1.13 (95 % CI = 1.10–1.16), with 0.95 % of results below the LOD; the GM for blood cadmium was 0.25 (95 % CI = 0.24–0.25), with 44.7 % below the LOD (Table 2). For the association between *T. gondii* seropositivity and metal concentrations (Figs. 2 and 3), the adjusted odds ratios associated with a doubling of blood lead and cadmium were 1.19 (95 % CI = 1.12–1.28) and 1.06 (95 % CI = 0.96–1.18), respectively (Table 3). There was a significant trend (*p* < 0.0001) in increasing seroprevalence by quartiles of lead (Table 3). The association between lead and *T. gondii* seropositivity was slightly weaker among children under 13 years old (Table 5, AOR = 1.12; 95 % CI = 0.84–1.49, per doubling of blood lead) compared to those 13–35 years old (AOR = 1.25; 95 % CI = 1.12–1.40) and those over 35 years old (AOR = 1.21; 95 % CI = 1.07–1.37) (Additional file 1, Tables S1 and S2). Lead and cadmium concentrations were only slightly correlated (Pearson’s *r* = 0.24), and no evidence was found to suggest additive or multiplicative interaction between lead and cadmium exposure in association with *T. gondii* seropositivity (Table 6).

**Table 6:** Effect of interaction between blood lead and blood cadmium levels on odds of *T. gondii* seropositivity, NHANES 1999–2004, 2009–10

Blood lead levels	Blood cadmium levels				
	<Median		≥Median		AOR (95 % CI) within Pb strata
	N (Weighted %)	AOR (95 % CI)	N (Weighted %)	AOR (95 % CI)	
<Median	4629 (5.2)	1.0	3892 (9.3)	1.24 (0.978–1.59)	1.26 (0.94–1.69
				*P* = 0.0805	*P* = 0.1222
≥Median	4101 (10.4)	1.45 (1.17–1.79)	5803 (17.0)	1.64 (1.30–2.07)	1.34 (0.97–1.85)
		*P* = 0.0007		*P* = <0.0001	*P* = 0.0779
AOR (95 % CI) within Cd strata		1.13 (0.78–1.62)		1.41 (1.22–1.63)	
		*P* = 0.5244		*P =* <0.0001	

Measure of interaction on additive scale: RERI (95 % CI) = -0.05 (−0.42–0.32)

Measure of interaction on multiplicative scale: ratio of ORs (95 % CI) = 0.91 (0.69–1.21) *P* = 0.5262

AORs are adjusted for NHANES cycle, age, gender, race/ethnicity, country of birth origin, family income, self-reported general health condition, and household crowding

### Hepatitis B virus

After excluding HBV-vaccinated participants and those with unknown vaccine status, exposure and HBV serology data were available for 17,389 non-smoking participants ≥6 years old from 7 NHANES cycles (1999–2012) (Fig. 1c). Overall seroprevalence for anti-HBc was 4.9 %. Seroreactivity significantly varied by age, gender, race, family income level, country of birth origin, general health condition, source of home tap water, crowded housing, and using illegal/illicit injectable drugs (Table 1). The blood lead GM concentration for all participants was 1.41 (95 % CI = 1.38–1.44), with 0.5 % of results below the LOD; the GM for blood cadmium was 0.30 (95 % CI = 0.30–0.31), with 26.4 % below the LOD (Table 2). In examining the association between anti-HBc seropositivity and blood concentrations (Figs. 2 and 3), the adjusted odds associated with a 2-fold increase (doubling) in blood levels of lead and cadmium were 1.19 (95 % CI = 1.08–1.32) and 1.38 (95 % CI = 1.23–1.55) respectively (Table 3). There were significant trends in increasing seroprevalence by percentiles of lead (p = 0.0077) and cadmium (*p* < 0.0001) (Table 3). Lead and cadmium concentrations were only slightly correlated (Pearson’s *r* = 0.31), and no evidence was found to suggest additive or multiplicative interaction between lead and cadmium exposure in association with HBV seropositivity (Table 7).

**Table 7:** Effect of interaction between blood lead and blood cadmium levels on odds of Hepatitis B seropositivity, NHANES 1999–2012

Blood lead levels	Blood cadmium levels				
	<Median		≥Median		AOR (95 % CI) within Pb strata
	N (Weighted %)	AOR (95 % CI)	N (Weighted %)	AOR (95 % CI)	
<Median	4160 (2.2)	1.0	3622 (4.5)	1.51 (1.12–2.04)	1.42 (1.03–1.98)
				*P*	*P* = 0.0310
≥Median	3011 (4.0)	1.23 (0.86–1.76)	6596 (7.9)	1.98 (1.51–2.61)	1.67 (1.23–2.25)
		*P* = 0.2503		*P* = <0.0001	*P* = 0.0008
AOR (95 % CI) within Cd strata		1.12 (0.79–1.60)		1.35 (1.06–1.72)	
		*P* = 0.5209		*P* = 0.0155	

Measure of interaction on additive scale: RERI (95 % CI) = 0.24 (−0.28–0.76)

Measure of interaction on multiplicative scale: ratio of ORs (95 % CI) = 1.07 (0.71–1.61); *P* = 0.7622

AORs are adjusted for age, gender, race/ethnicity, country of birth origin, family income, self-reported general health condition, use of illicit/street injected drugs

We considered serum iron in all analyses, however, we excluded it from the final models because it was statistically insignificant in all models, did not impact the coefficients of lead and cadmium, and reduced the effective sample size due to missing data.

## Discussion

We observed statistically significant increased odds of elevated blood lead and cadmium levels among non-smoking NHANES participants seropositive for *H. pylori, T. gondii*, and HBV*.* The associations between lead and cadmium exposures and *H. pylori* seropositivity were strongly pronounced among participants under 13 years of age, particularly for lead. For each of the three chronic infections, non-smoking participants had an approximately 20 % increase in odds of being seropositive for each doubling of blood lead levels (Table 3). For HBV and *H. pylori* infections, a doubling of blood cadmium levels corresponded to nearly a 40 % increase in odds of being seropositive (Table 3). We found no evidence of additive or multiplicative interaction between lead and cadmium. These results support the growing evidence that heavy metal exposure has deleterious effects on the immune response.

Suppression of the immune response may lead to increased susceptibility of the host to chronic infection. In addition to an immunotoxic effect, heavy metals may also influence the severity of infection. An *in vitro* study found that cadmium-induced oxidative stress increased influenza virus replication in Madin-Darby canine kidney (MDCK) cells [31]. An experimental study in mice suggested that during a Coxsackievirus B3 infection, levels of mercury flux between tissues such that higher levels were retained in infected organs [32]. Another mouse study reported that pretreatment with a single dose of cadmium or manganese increased the severity of symptoms and mortality to sub-lethal viral infections, including Venezuelan Equine Encephalitis virus [33].

Our analysis contributes to the growing body of literature that has used NHANES data to examine associations between heavy metal exposures and infections. Previous NHANES analyses have reported associations between chronic HBV infections and blood mercury levels in women of child-bearing age [34], higher blood cadmium levels among HIV-infected individuals [35], elevated blood lead levels and an increased risk of herpes simplex virus type 2 infections [36], as well as an inverse association between urinary arsenic and Varicella Zoster virus seroprevalence [37].

HBV is a bloodborne virus that attacks the liver. HBV pathogenesis results from interactions between the virus and the host’s immune system. Immunosuppression of the host can result in chronic HBV infections. Lead and cadmium are hepatotoxic [38, 39] and have been shown to increase viral activity [31, 40]. In addition, dietary cadmium has been shown to enhance the progression of hepatocellular tumors in HBV-infected mice [41]. A recent study in China found decreased HBV surface antibody (HBsAb) levels among preschool children exposed to lead from electronic and electrical waste (e-waste) [42]. The authors reported almost 50 % of children with chronic lead exposure failed to develop sufficient immunity against HBV in response to vaccination; therefore, their immune systems may be more susceptible to HBV infection in the future. Another study conducted in Babylon found blood levels of lead to be significantly higher in HBV patients compared to healthy controls [43].

*T. gondii,* an obligate intracellular protozoan, is one of the most widespread zoonotic parasites in the world [44]. When oocysts are ingested by an intermediate host such as humans, they migrate in the host’s body and form permanent cysts in various organs and tissues [45]. Toxoplasmosis is lifelong infection that has the potential to reactivate and cause severe disease and even death in immunocompromised individuals. Existing data on the effect of lead and cadmium on chronic toxoplasmosis are lacking; however, lead has been suggested to inhibit intracellular killing of Leishmania parasites [46]. Although age-specific associations between blood lead and *T. gondii* seropositivity were relatively similar, among children under 13 years old, the association was slightly weaker compared to older age groups. One of the most common routes of transmission for *T. gondii* to humans in the United States is suspected to be raw meat consumption [45]; therefore, in addition to having more opportunities to be infected as one ages, eating habits in adulthood may also contribute to this observed association with older age.

Half of the world's population is infected with *H. pylori* [47]. The bacterium colonizes the inner mucus layer of the stomach epithelium, but the majority of infections remain asymptomatic. Up to 20 % of *H. pylori* infections lead to severe chronic disease outcomes, including chronic gastritis, peptic ulcer, gastric mucosa-associated lymphoid tissue (MALT) lymphoma, and gastric cancer [48–51]. While there are no published data describing the association between lead and cadmium and chronic helicobacteriosis, researchers have suggested that maintaining metal homeostasis, including cadmium levels, is critical for *H. pylori* to adapt to and survive in the gastric environment [52].

The stronger association between *H. pylori* seropositivity and lead and cadmium exposures among children may be due to an increased vulnerability or susceptibility, due to increased and faster absorption of these metals during a time when hygienic factors may also make them more likely to be exposed to *H. pylori.* Although we controlled for numerous available factors related to socioeconomic status and living conditions (income, crowding), these associations could also reflect a shared environmental susceptibility that we were unable to fully control for in our analysis.

We found no evidence to suggest either additive or multiplicative interaction between elevated lead and cadmium levels and their association with seropositivity. Lead and cadmium share common environmental exposure routes through contaminated food, water, and soil. Individual toxic metals can interact with toxic and essential metals in various tissues [53]. Mixed-metal exposure may yield synergistic interactions, or even novel effects not seen in single metal exposures [54, 55]; however, information on the interaction of low-dose toxic metal mixtures is lacking [5, 56]. Studies have reported that exposure to lead increased the renal response to low levels of cadmium [57], and a dose-dependent interaction between prenatal co-exposure to lead and cadmium has been observed [58]. Because lead and cadmium have common, as well as distinctive, binding sites and mechanisms of action, the pathophysiological effects caused by each metal alone may be exacerbated during co-exposure [59]. It remains unclear whether our lack of evidence for interaction was because synergistic interaction does not influence immunomodulatory effects or because our method of examining co-exposure was inadequate. Furthermore, including both lead and cadmium levels as covariates in a single multivariable model (rather than separately in individual models as reported here), did not significantly change the observed effects (data not shown).

Continuous NHANES collects valuable health information from a national, representative sample of the United States population, making it an effective tool for hypothesis generation and exploring biomarker data for chemical risk assessments [60]. Nonetheless, we recognize the inherent limitations of its cross-sectional design that prevent us from inferring causality. In addition, combining datasets across multiple survey years can be a limitation, depending on the covariates of interest. Questions and medical exams are not continuous across the survey years. In addition, several questionnaires and metal exposure assessments are administered to only subgroups (e.g. adults ≥20 years old, females of child-bearing age, or a special one-third subsample of adults). Our goal was to maintain the largest cohort as possible for consistency and increased power. Despite these limitations, this study provides risk factor data that can inform future environmental assessments and prospective human studies to better link cause and effect for heavy metal exposure and risk of chronic infections.

## Conclusions

Environmental exposure to lead and cadmium, even at chronic low-level doses, remains a significant public health concern [54]. The results of this cross-sectional human health survey suggest that the immunological effects of lead and cadmium toxicity may result in an increased susceptibility to chronic infections. While the mechanisms of immunotoxicity vary by metal type, exposure dose, temporality to infection, severity of infection, and genetic factors [1, 54, 61, 62], increased environmental contamination with heavy metals will likely contribute to an increased incidence of chronic infections in human populations.

## Additional file

Additional file 1:
**Supplemental Tables.** Description of data: Seropositivity associated with blood lead and cadmium levels among non-smoking NHANES participants, by age groups 13-35 and 35 and over. (PDF 238 kb)
