# Supplementary material for: Elevated blood lead and cadmium levels associated with chronic infections among non-smokers in a cross-sectional analysis of NHANES data
Source: Environ Health. 2016 Feb 11;15:16. doi: 10.1186/s12940-016-0113-4 (PMC4750187; doi:10.1186/s12940-016-0113-4)
Supplement: Additional file 1: — Supplemental Tables. Description of data: Seropositivity associated with blood lead and cadmium levels among non-smoking NHANES participants, by age groups 13-35 and 35 and over. (PDF 238 kb) [file 12940_2016_113_MOESM1_ESM.pdf]

Supplemental Tables

Table S1. Seropositivity associated with blood lead and cadmium levels, for each two-fold increase and across percentiles, among non-smoking NHANES participants, 1999-2012. Ages 13-35 years.

**Heavy metal**		***H. pylori***		***T. gondii***	
		**Positive (Weighted %)**	**AOR (95% CI)[a,b]**	**Positive (Weighted %)**	**AOR (95% CI)[a,c]**
Per doubling of blood lead		18.6	**1.57 (1.18-2.09)**[d]	9.3	**1.25 (1.12-1.40)**
Blood lead concentration (µg/dL)					
	Quartile 1	10.1	Ref	5.2	Ref
	Quartile 2	17.5	1.62 (0.96-2.73)	7.2	1.26 (0.91-1.75)
	Quartile 3	23.5	**2.03 (1.17-3.52)**	11.3	**1.72 (1.26-2.36)**
	Quartile 4	42.9	**3.49 (1.75-6.98)**	19.1	**1.99 (1.44-2.74)**
	p for trend	0.0001		<0.0001	
Per doubling of blood cadmium		18.6	**1.81 (1.29-2.55)**	9.3	1.04 (0.87-1.25)
Blood cadmium concentration (µg/L)					
	Tertile 1	11.6	Ref	6.0	Ref
	Tertile 2	21.8	**2.08 (1.52-2.86)**	9.4	1.18 (0.80-1.74)
	Tertile 3	25.4	**2.04 (1.27-3.29)**	11.3	1.30 (0.93-1.82)
	p for trend	0.0034		0.43	

[a]Multivariable logistic regression used with survey procedures
[b]Adjusted for age, gender, race/ethnicity, country of birth origin, family income, self-reported general health condition, tap water source, and household crowding.
[c]Adjusted for NHANES cycle, age, gender, race/ethnicity, country of birth origin, family income, self-reported general health condition, and household crowding.
[d]Bolded font denotes statistically significant (α< 0.05).

Table S2. Seropositivity associated with blood lead and cadmium levels, for each two-fold increase and across percentiles, among non-smoking NHANES participants, 1999-2012. Over 35 years of age.

**Heavy metal**	***H. pylori***		***T. gondii***	
	**Positive (Weighted %)**	**AOR (95% CI)[a,b]**	**Positive (Weighted %)**	**AOR (95% CI)[a,c]**
Per doubling of blood lead	31.7	1.10 (0.94-1.28)	15.6	**1.21 (1.07-1.37)**[d]
Blood lead concentration (μg/dL)				
Quartile 1	24.7	Ref	9.0	Ref
Quartile 2	28.4	1.06 (0.61-1.84)	13.0	1.17 (0.78-1.77)
Quartile 3	30.4	1.13 (0.71-1.78)	17.2	1.55 (1.05-2.29)
Quartile 4	37.5	1.18 (0.70-2.00)	18.5	1.44 (0.98-2.10)
p for trend	0.43		0.027	
Per doubling of blood cadmium	31.7	1.22 (0.96-1.89)	15.6	1.08 (0.95-1.23)
Blood cadmium concentration (μg/L)				
Tertile 1	25.0	Ref	12.0	Ref
Tertile 2	28.2	0.96 (0.56-1.65)	13.7	1.18 (0.80-1.74)
Tertile 3	34.4	1.06 (0.71-1.58)	17.5	1.30 (0.93-1.82)
p for trend	0.72		0.092	

[a]Multivariable logistic regression used with survey procedures
[b]Adjusted for age, gender, race/ethnicity, country of birth origin, family income, self-reported general health condition, tap water source, and household crowding.
[c]Adjusted for NHANES cycle, age, gender, race/ethnicity, country of birth origin, family income, self-reported general health condition, and household crowding.
[d]Bolded font denotes statistically significant ($\alpha < 0.05$).
